# Comparative assessment of a novel fan box trap for collecting *Anopheles farauti* and culicine mosquitoes alive in tropical north Queensland, Australia

**DOI:** 10.1093/jme/tjad156

**Published:** 2024-01-17

**Authors:** Weng K Chow, Robert D Cooper, Matthew Lokhorst, Myron P Zalucki, Luke Ambrose, Nigel W Beebe

**Affiliations:** Australian Defence Force Malaria and Infectious Disease Institute, Enoggera, Queensland, Australia; Australian Defence Force Malaria and Infectious Disease Institute, Enoggera, Queensland, Australia; Australian Defence Force Malaria and Infectious Disease Institute, Enoggera, Queensland, Australia; School of the Environment, The University of Queensland, St Lucia, Queensland, Australia; School of the Environment, The University of Queensland, St Lucia, Queensland, Australia; School of the Environment, The University of Queensland, St Lucia, Queensland, Australia

**Keywords:** mosquito trap, surveillance, mosquito survival, trap comparison

## Abstract

During preliminary mosquito surveys at Cowley Beach Training Area in north Queensland, Australia, it was found that the utility of the standard encephalitis virus surveillance (EVS) trap for collecting the malaria vector *Anopheles farauti* (Laveran) adults was compromised by the harsh tropical conditions. With the aim of increasing the survival rate of mosquitoes, we designed a downdraft fan box trap (FBT) that incorporated a screened fan at the bottom of the trap, so mosquitoes did not have to pass through a fan. The FBT was tested against the EVS and Centers for Disease Control (CDC) light traps, where mosquitoes do pass through a fan, and a nonpowered passive box trap (PBT). We conducted 4 trials to compare the quantity and survival of *An. farauti* and culicine mosquitoes were collected in these traps. Although not significant, the FBT collected more *An*. *farauti* than the EVS trap and PBT and significantly less *An. farauti* than the CDC light trap. However, the FBT improved on the CDC light trap in terms of the survival of *An*. *farauti* adults collected, with a significantly higher percentage alive in the FBT (74.6%) than in the CDC light trap (27.5%). Thus, although the FBT did not collect as many anophelines as the CDC, it proved to be superior to current trap systems for collecting large numbers of live and relatively undamaged mosquitoes. Therefore, it is recommended that FBTs be used for collecting *An. farauti* adults in northern Australia, especially when high survival and sample quality are important.

## Introduction

Throughout the Southwest Pacific region, malaria occurrence has a wide distribution, being found from the Moluccas (Indonesia) in the west to Papua New Guinea (PNG), Solomon Islands, and Vanuatu (Beebe et al. 2013, World Health Organization 2021). This is a major concern for the Australian Defence Force (ADF) when deploying soldiers to this region for training and humanitarian aid efforts (Shanks 2017). The primary malaria vector in these countries is *Anopheles farauti* Laveran, 1902 (Cooper and Frances 2002, Cooper et al. 2002, 2009, Beebe et al. 2015). This species is prevalent in northern Australia (Cooper et al. 1996) and was responsible for the past outbreaks of malaria in this region (Black 1972, Harley et al. 2001, Hanna et al. 2002, Cooper et al. 2009, Knope et al. 2014).

*Anopheles* mosquitoes are often collected through trapping to study species composition, vector biology, ecology, and to identify the pathogens they carry. This knowledge can contribute to the planning and assessment of intervention strategies against mosquito-borne diseases. Two of the more commonly used mosquito traps for collecting questing adults are the CO_2_-baited encephalitis virus surveillance (EVS) trap (Rohe and Fall 1979) and Centers for Disease Control (CDC) light trap (Sudia and Chamberlain 1962), both of which use an electric fan at the entrance to draw mosquitoes through the fan blades into a collection container. The Australian Defence Force Malaria and Infectious Disease Institute (ADFMIDI, formerly the ADF-Army Malaria Institute) has extensively used EVS traps in malaria and arbovirus vector surveys in tropical regions including northern Australia and Papua New Guinea (Sweeney et al. 1990, Cooper et al. 1996, 2004, Cooper and Frances 2002, Frances et al. 2004).

There are a number of issues with using these traps in tropical regions, which are characterized by high temperatures, humidity, and rainfall. Excessive moisture, either from intense rainfall or condensation from the dry ice container which serves as a carbon dioxide (CO_2_) source, often results in problems with the power supply and motors failing. The ingress of moisture into the collection container also reduced the quality of the mosquitoes collected, affecting morphological based species identification, DNA quality, and subsequent biological studies, which require mosquitoes to be alive and in good condition. Other CO_2_-baited light trap issues include the limited availability of batteries in some locations, high maintenance, and component replacement costs.

In attempts to overcome some of these problems with powered traps, various nonpowered CO_2_-baited passive traps that operate without a fan (Schreck 1970, Ritchie et al. 2013, Van de Straat et al. 2019) have been designed, eliminating problems with electronic components, motors, and the need to recharge or replace batteries. One study (Schreck 1970) found that a passive plexiglas trap was effective in collecting large numbers of culicines but not anophelines. In another study in northern Australia, Ritchie et al. (2013) demonstrated that CO_2_-baited passive traps collected a comparable number of mosquitoes and, with some models, more mosquitoes than the CO_2_-baited CDC light trap.

Most mosquito trap evaluation studies have focused on how trap type affects mosquito species richness, distribution, and abundance (Carestia and Savage 1967, Ritchie and Kline 1995, Van Den Hurk et al. 1997, Cooper et al. 2004, Tangena et al. 2019), but few studies have investigated the quality of the mosquitoes collected in the traps. Previous studies have observed that a percentage of the adult mosquito catch in powered traps were too damaged to be identified by morphology (Vanessen et al. 1994, Frances et al. 2004, Overgaard et al. 2012, Van de Straat et al. 2019, Cansado-Utrilla et al. 2020). In contrast, mosquitoes collected in passive traps do not go past fan blades, so should be relatively undamaged. To aid our ongoing studies of the malaria vector *An*. *farauti* in the Southwest Pacific, we developed a rain-proof fan box trap (FBT) system and compared the quantity and quality of mosquitoes collected with EVS and CDC light traps, and the nonpowered passive box trap (PBT; Ritchie et al. 2013) systems.

## Materials and Methods

### Study Site

**Fig. 1.**
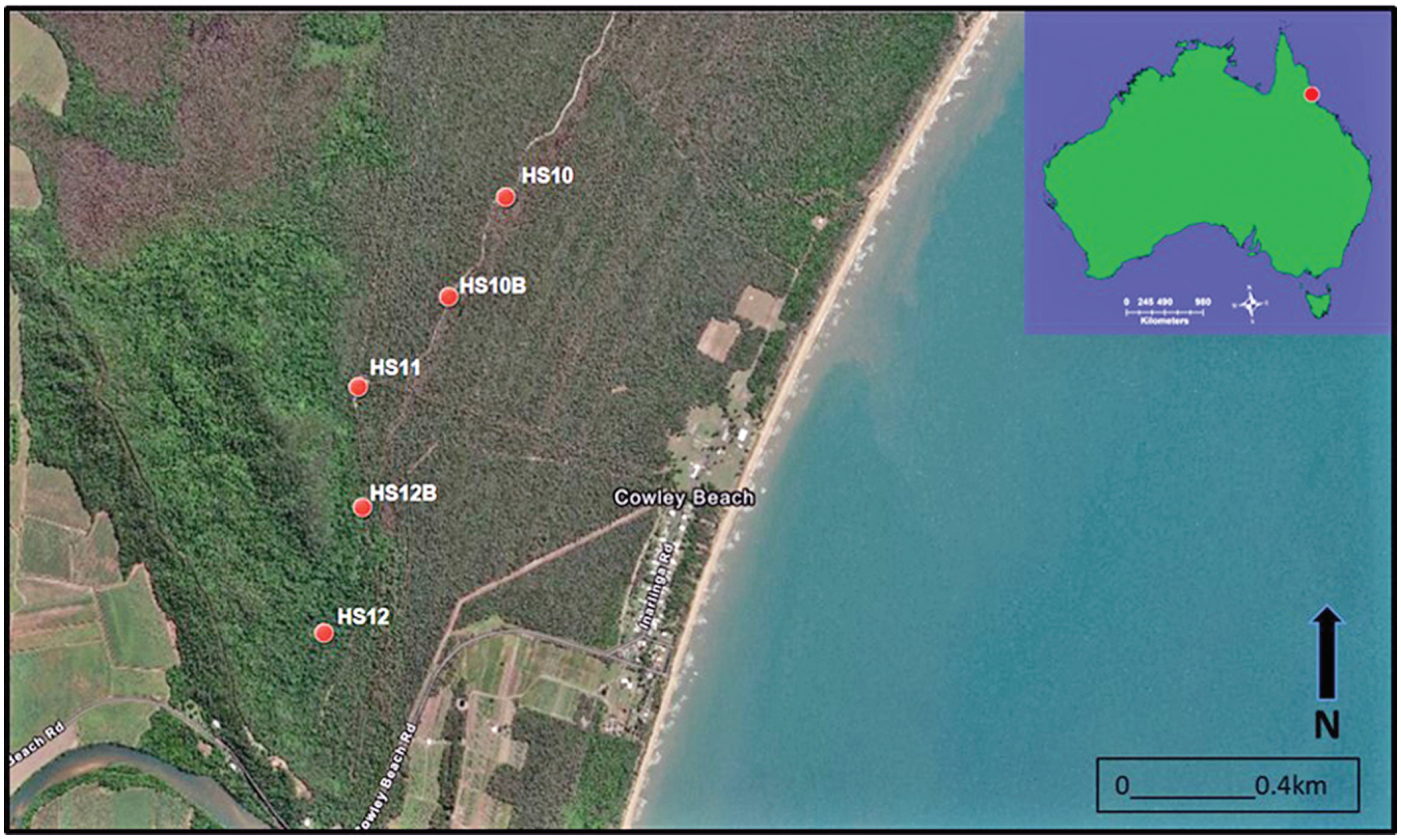
Mosquito trap evaluation sites at Cowley Beach Training Area, Queensland, Australia. Sites HS10 and HS10B were in open forest; site HS11 was at the boundary of rainforest and open forest and sites HS12 and HS12B were located within rainforest.

The study was conducted in 2015 at Cowley Beach Training Area (CBTA) (17°41ʹ35″ S, 146°6ʹ40″ E) which is located within the wet tropics region of north Queensland, Australia. The climate in the region is monsoonal with distinct wet and dry seasons. The area receives a mean annual rainfall of 3,500 mm (Australian Bureau of Meteorology 2022). The annual dry season (May to October) receives a mean rainfall range from 85 to 302 mm, and the wet season (November to April) receives a mean rainfall range from 156 to 663 mm (Australian Bureau of Meteorology 2022). The mean minimum and maximum temperatures in the wet season were between 19.4 and 30.9°C, respectively, and in the dry season were between 15.3 and 28.4°C, respectively (Australian Bureau of Meteorology 2022). The mean relative humidity was high (70–80%) and constant throughout the year. The rainfall, humidity, and temperature were recorded for each night of the trial using a Davis Vantage Vue weather station (Model#: 6357, Davis Instrument Corporation, California, USA). Mosquito traps were evaluated at 5 sites within the CBTA with each site separated by 400 m (Fig. 1). Sites HS10 and HS10B were in low to medium open forest and bushland; site HS11 was at the boundary of tall rainforest and medium open forest; and sites HS12 and HS12B were located within tall wet sclerophyll rainforest. Trap sites with different height vegetation and foliage density were selected to reduce bias on environmental characteristics that may influence mosquito catch numbers across different landscapes.

### Traps Used

**Fig. 2.**
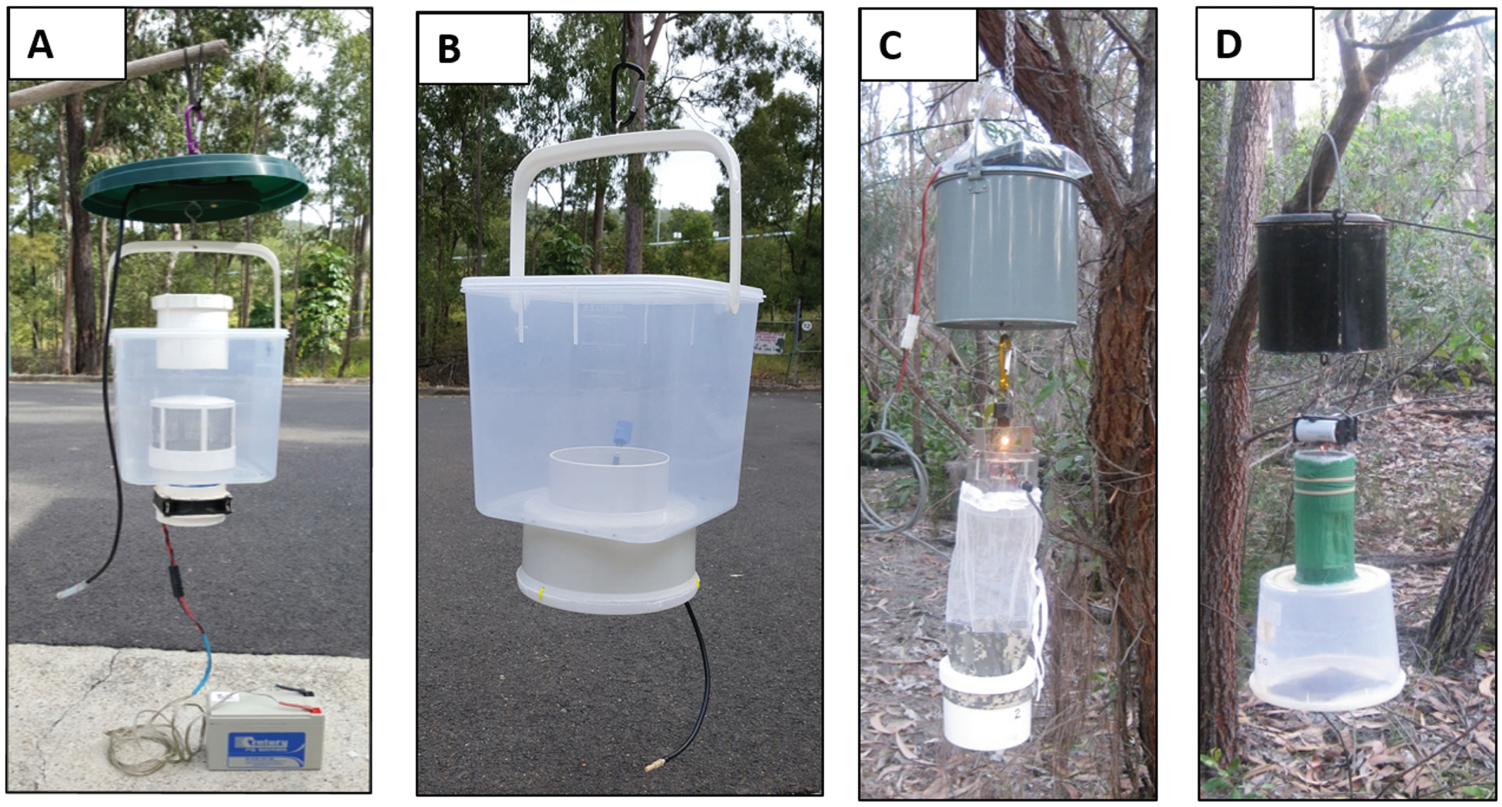
Images of the fan box trap (A), passive box trap (B), Center for Disease Control light trap (C), and encephalitis virus surveillance trap (D) used in comparison trials in 2015 at Cowley Beach Training Area, Queensland, Australia.

The EVS light trap (Discontinued, Model: EVS CO_2_ Mosquito Trap, Australian Entomological Supplies Pty Ltd, New South Wales, Australia) and the CDC light trap (Model#: 512, John W. Hock Company, Florida, USA) were used as per manufacturer recommendations except that the standard collection net on the EVS was replaced by a 5-liter plastic bucket and we followed the method used by the ADF, which did not deploy the CDC light trap with the lid (Fig. 2). Both of these traps are down draft traps with the motor and fan mounted above the entrance to the collection container. Both traps were deployed with CO_2_, in the form of 700 g dry ice, as an attractant. The dry ice was placed in a 4-liter insulated container situated directly above the fan housing; as the dry ice sublimated, it was released from four 3-mm-diameter holes at the bottom of the container. The remaining dry ice was weighed at the end of the collection period to determine the amount of dry ice used so that the CO_2_ release rate could be estimated. The dry ice CO_2_ output for the EVS and CDC light trap during the trial was estimated to be 530 ml/min. The EVS trap was powered by two 1.5V D cell batteries mounted directly above the fan; the CDC light trap was powered by four 1.5V D cell batteries adjacent to the trap and connected to the motor by a lead and both these batteries were replaced every 24 h.

The PBT (Model#: GPT009, culicid-traps@netspeed.com.au, Fig. 2) consisted of an 8.5-liter plastic collection box with a removable lid. Friction mounted to the base of the collection box was a 100-mm-diameter polyvinyl chloride (PVC) spigot adapter. Inside the spigot adapter was attached a 150-mm-diameter stainless steel wire dome sieve with a 50-mm entry hole in the center. Compressed CO_2_ gas (Gas code#: 082, BOC, Queensland, Australia) was supplied to the trap via a length of 5-mm-diameter plastic tubing ending with an aquarium stone for CO_2_ diffusion; this was inserted into the catch box and affixed on the PVC spigot adapter and the released CO_2_ gas exited from the sieve and entry hole. The 5-mm plastic tubing was attached to an adjustable gas regulator (Model#: CO_2_ regulator, culicid-traps@netspeed.com.au) connected to a CO_2_ gas bottle to enable controlled release of CO_2_ gas. The PBT was suspended from a tree branch via a carabiner attached to the box handle.

**Fig. 3.**
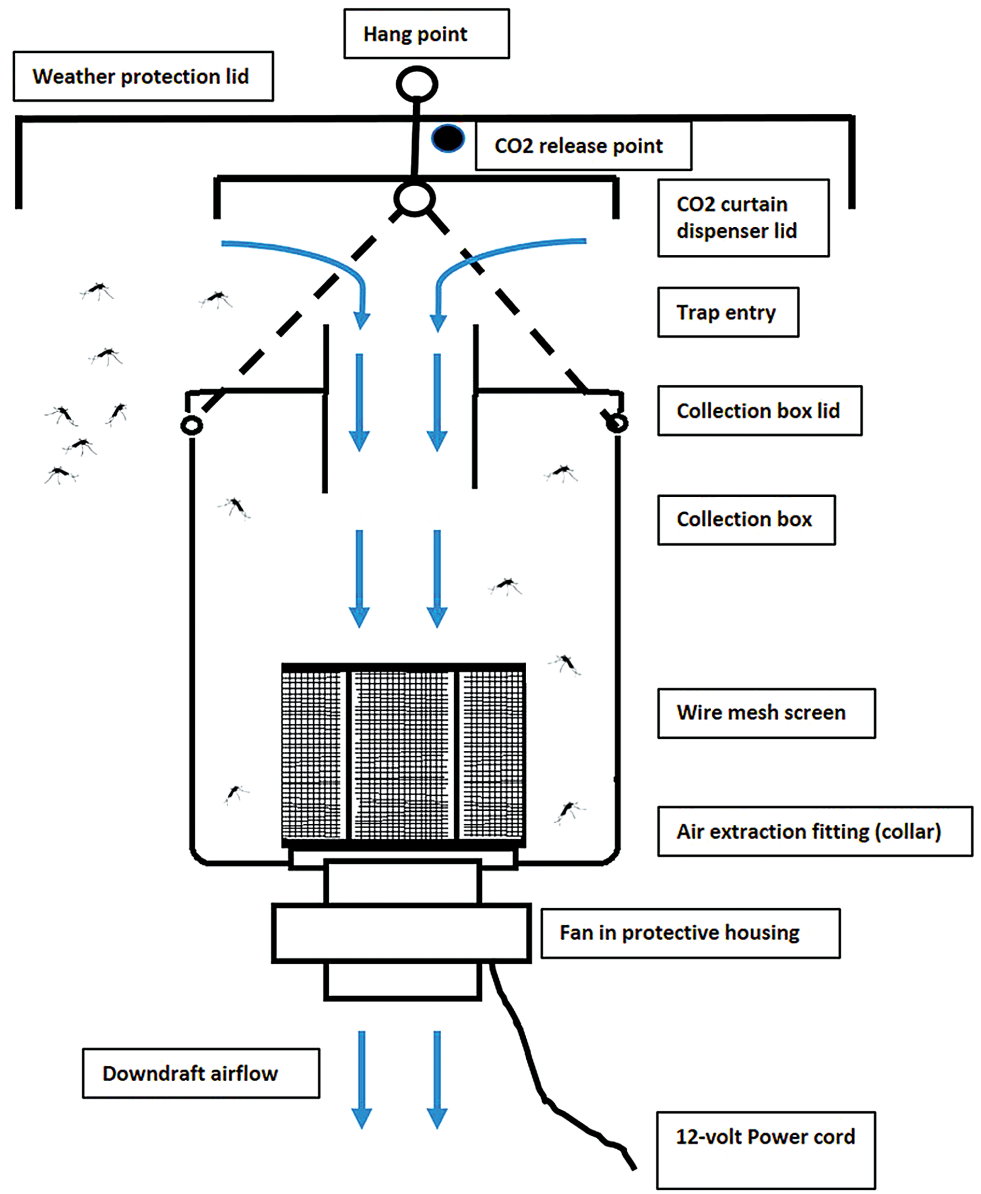
Fan box trap schematic. The arrows refer to the direction of airflow.

The basic design of the FBT (Figs. 2 and 3) can be regarded as a down draft trap; mosquitoes are lured to the top of the trap with CO_2_ and drawn down into the trap by a down draft created by a fan mounted at the base of the trap. The trap consisted of a 300-mm-diameter weather protection lid under which was suspended a plastic 8.5-liter collection box (Item#: 105900, The Décor Corporation Pty Ltd, Victoria, Australia). A 90-mm-diameter hole was cut into the top of the collection box lid (entry point), and a second hole into the bottom of the collection box base (air extraction point). A section of PVC piping (90 mm diameter × 80 mm long) was inserted halfway (40 mm) into the entry point hole. A 90-mm PVC-threaded coupling was then slid over the original piece of pipe from the outside and a 90-mm nonthreaded coupling from the inside and these were clamped to the top of the collection box lid and adhered with PVC glue; silicon sealant was also applied on the outside and inside of this joint for additional waterproofing. To the top of the 90-mm threaded coupling entry point was a screw-on 90-mm PVC-threaded cap for sealing the trap at the end of the collection period. A 100 × 90 mm PVC socket reducer collar was inserted into the hole at the air extraction point. On the inside of the collection box, a 0.95-mm-wire mosquito mesh cowl (Model#: TAVC01, Rain Harvesting, Queensland, Australia) was friction mounted to the 90-mm side of the collar and was detachable. On the outside of the collection box, a 12V DC computer cooling fan (Model#: Duratech YX2572, Jaycar, Queensland, Australia) in a protective housing was friction mounted to the 100-mm-diameter side of the collar and both were detachable. The fan was powered by a 12V, 7 amp sealed lead acid rechargeable battery connected by a lead to the fan motor. These batteries operate for 72 h before requiring a recharge. The collection box was suspended from a tree branch via a carabiner attached to the box handle, below the weather protection lid. Compressed CO_2_ gas was supplied to the trap via a length of 5-mm-diameter plastic tube ending with an aquarium stone, this was mounted above the entry point between the weather protection lid and a smaller 170-mm plastic disc so that the released gas fell as a curtain around the entry point of the trap. The 5-mm plastic tubing was attached to an adjustable gas regulator (Model#: CO_2_ regulator, culicid-traps@netspeed.com.au) connected to a CO_2_ gas bottle to enable control release of CO_2_ gas. During the trials, an anemometer (Model#: KC-280A, NDI Tools Pty Ltd, Victoria, Australia) determined the entry point suction air flow rate for the FBT, EVS, and CDC light trap was 3.5, 3.1, and 3.1 m/s, respectively. The CO_2_ gas release rate for both the FBT and PBT was set at 500 ml/min.

### Mosquito Quantity Comparisons

Three Latin square design field trials were conducted (Cochran 1992). In this design, traps were rotated each night within each trap site to reduce day (trap night) and trap position bias. The first trial (April 12–17) compared mosquito quantity (primarily *An*. *farauti*) collected in the standard EVS trap with the PBT. This trial was conducted for four nights at site HS11. The second trial (April 18–19) compared the EVS trap and the FBT over 2 nights at sites HS10 and HS11. A third trial (October 7–14) using the FBT, EVS trap, and CDC light trap was studied for 6 nights at all 5 sites. All traps were set so the base of collection boxes was 1 m above the ground and the traps at each site were separated by 20 m. The traps were set at 1800 h and collected at 2400 h. At the end of each collection period, the mosquitoes collected were killed by placing the collection containers into a −20°C freezer. The collections were then sorted into anophelines and culicines and counted. For field trials 1 and 2, we assessed whether there are statistically significant differences between the number of mosquitoes collected in each trap type by performing Wilcoxon tests using the “wilcox_test” function in R (R-Core-Team 2017). For field trial 3, we used a Kruskal–Wallis test, also performed in R using the “kruskal.test” function. To assess the significance of differences between specific trap types, we then used pairwise Wilcoxon tests with Bonferroni corrections for multiple comparisons in R using the “pairwise.wilcox.test” function. These comparisons were performed on total catch as well as on anopheline and culicine mosquitoes separately.

### Mosquito Survival Comparisons

The survival of mosquitoes collected in the FBT and CDC light traps was evaluated from 15 to 19 October 2015. The study design was similar to the trap quantity comparison; however, only sites HS10, HS11, and HS12 were used, and the collections were run over 5 nights. At the end of each collection period, the mosquitoes were classified as either alive or dead for anophelines and culicines separately; alive mosquitoes were those that could still fly, while dead mosquitoes were those lying in the bottom of the collection box regardless of whether they could move or not. After the number of mosquitoes alive or dead was counted, all the mosquitoes were killed by freezing, and the total number collected was counted. The number dead was determined by subtracting the number alive from the total number. It was not possible to count directly from the collection of the CDC light traps, so the container was opened inside a mosquito cage (30 × 30 × 30 cm Perspex box with a mosquito mesh opening on 1 side) and the numbers alive and dead were then counted. To assess whether there was a significant difference in the number of mosquitoes surviving in the CDC light traps and FBTs, we performed Fisher’s exact tests (Fisher 1934) to compare the proportion of living versus dead mosquitoes collected from each taxonomic group (anophelines and culicines) in each trap type, using the “fisher.test” function in R (R-Core-Team 2017).

### Molecular Analyses

All anophelines collected were initially identified as *An. farauti* based on morphology. However, 2 other isomorphic *Anopheles*, *Anopheles hinesorum* Schmidt, 2001, and *Anopheles torresiensis* Schmidt, 2001, are known to occur in the region (Cooper et al. 1996). Subsamples of *An. farauti* from each of the 5 trap sites were assessed by polymerase chain reaction (PCR) to confirm that the target study species was *An. farauti*. Field-collected specimens were preserved in 70–100% ethanol for transport back to the laboratory where *Anopheles* specimens underwent individual salt extractions to obtain genomic DNA (Beebe et al. 2001) and species identification by a diagnostic PCR-restriction fragment length polymorphism based on Internal Transcribed Spacer region 2 (Beebe and Saul 1995).

## Results

During the study, a total of 52,993 mosquitoes were collected from all traps, consisting of 18,371 *An. farauti*, and 34,622 culicines. A subset of *An. farauti* from the 5 trap sites were confirmed to be *An. farauti* (*n* = 669) and no other *An. farauti* complex species were identified. For all trap trials, the culicine mosquito species comprised *Aedes kochi* (25%), *Coquillettidia xanthogaster* (25%), *Verrallina lineata* (10%), *Verrallina carmenti* (10%), *Aedes vigilax* (5%), *Aedes tremulus* (5%), *Culex annulirostris* (5%), and *Culex sitiens* (5%), while 10% of the collections were damaged and unidentifiable.

For both trials 1 and 2 (Table 1), the difference in the number of mosquitoes collected was not significant (Wilcoxon test: *P* > 0.05), although traps with a motorized fan collected over twice as many mosquitoes as nonpowered passive traps during trial 1. In trial 2, although not significant, the FBT outperformed the EVS trap in the total collection of both *An*. *farauti* and culicines by 61% and 37%, respectively (Table 1). In trial 3, there was a significant difference in the number of anophelines collected in the 3 different trap types (Kruskal–Wallis chi-squared = 11.4, df = 2, *P* = 0.003). However, there was no significant difference in either the overall number of mosquitoes (Kruskal–Wallis chi-squared = 5.62, df = 2, *P* = 0.06) or the number of culicines caught by the 3 trap types (Kruskal–Wallis chi-squared = 2.56, df = 2, *P* = 0.28). In this trial, the CDC traps collected significantly more *An. farauti* than both the FBT (Bonferroni-corrected Wilcoxon test, *P* = 0.0124) and EVS traps (Bonferroni-corrected Wilcoxon test, *P* = 0.005) (Table 1). The numbers of *An. farauti* collected by the EVS traps and FBTs were not significantly different (Bonferroni-corrected Wilcoxon test, *P* = 0.6466). Since the FBT and CDC light traps consistently collected more mosquitoes than the EVS traps, the FBT and CDC light traps were selected to evaluate the survival of mosquitoes collected. Survival rates were significantly higher in FBTs than the CDC light traps for both anophelines (Fisher’s exact test: *P* < 0.001, odds ratio = 7.745, 95% confidence interval [CI]: 6.77, 8.87) and culicines (Fisher’s exact test: *P* < 0.001, odds ratio = 21.35, 95% CI: 18.94, 24.11) (Table 2).

**Table 1.** Mean ± SE of *Anopheles farauti* and culicines collected by CO_2_-baited fan box traps, Centers for Disease Control light traps, encephalitis virus surveillance traps, and passive box trap during each trial conducted at Cowley Beach Training Area, Queensland (*n* = number of mosquitoes collected over the trial sampling period)

Date	Species	FBT	CDC	EVS	PBT
**Trial 1** 2015 April 4 nights	*An. farauti*	—	—	549 ± 218 (n = 2,195)	223 ± 142 (n = 892)
	Culicines	—	—	556 ± 212 (*n* = 2,225)	176 ± 46 (*n* = 704)
**Trial 2** 2015 April 2 nights	*An. farauti*	1,236 ± 556 (*n* = 3,707)	—	480 ± 271 (*n* = 1,439)	—
	Culicines	609 ± 284 (*n* = 1,828)	—	382 ± 74 (*n* = 1,145)	—
**Trial 3** 2015 October 6 nights	*An. farauti*	54 ± 12 (*n* = 1,623)	89 ± 12 (*n* = 2,683)^*^	42 ± 8 (*n* = 1,279)	—
	Culicines	204 ± 24 (*n* = 6,125)	199 ± 24 (*n* = 5,971)	160 ± 19 (*n* = 4,805)	—

^*^Pairwise Wilcoxon tests with Bonferroni corrections: significantly more mosquitoes collected *P* < 0.05.

**Table 2.** Mosquito survival summary of *Anopheles farauti* and culicines collected in the CO_2_-baited fan box trap and Centers for Disease Control light trap during the 5 nights across 3 trap sites at Cowley Beach Training Area on 15–19 October 2015

Species	FBT			CDC		
	Alive	Dead	Total	Alive	Dead	Total
*An. farauti*	74.6%^*^	25.4%	2,026	27.5%	72.5%	2,527
Culicines	94.4%^*^	5.6%	6,474	44.0%	56.0%	5,345

Significantly more alive mosquitoes were caught in fan box traps than in Centers Disease Control light traps for both anopheline (*P* < 0.001, odds ratio = 7.745, 95% CI: 6.77, 8.87) and culicine mosquitoes (Fisher’s exact test: *P* < 0.001, odds ratio = 21.35, 95% CI: 18.94, 24.11) according to Fisher’s Exact Tests.

^*^ *P* < 0.001.

## Discussion

In this study, we present results from a trial that evaluated the FBT, which was designed to avoid mosquitoes passing through fan blades and to hold them in a weather-resistant collection container, thus increasing the survival of collected mosquitoes. On 3 of the test nights during trial 3, up to 12 mm of rainfall was recorded. This caused major issues with the catch containers of the EVS and CDC light traps because the catch containers were not as rain resistant as the FBT. Both the EVS and CDC light trap catch containers are attached to the fan motor via a mesh sleeve, which becomes a point of entry for water to enter the catch containers. Furthermore, condensation on the dry ice CO_2_ container can lead to the water dripping onto the fan motor and into the catch container, also causing water damage to the mosquito catch. This issue was exacerbated when the catch containers were placed into the freezer for mosquito immobilization, as mosquitoes became frozen in ice at the bottom of the container making morphological identification difficult when defrosted, as well as potentially impacting the DNA quality of specimens.

In both the EVS and CDC light traps, the mosquitoes are drawn into the catch container through the fan blades often resulting in mosquito damage. This observation was also noted in other studies (Sandoski et al. 1983, Van de Straat et al. 2019). Fan placement at the base of the FBT resulted in a higher survival rate compared with the CDC trap. This is likely due to mosquitoes being drawn into the collection box through the top entry hole without passing through fan blades limiting damage to trapped mosquitoes. Indeed, during the sorting of trap collections, it was noted that the EVS and CDC light traps contained damaged specimens (i.e. decapitation of heads, loss of body scales, and wing damage).

No light was used for the FBT because the presence of a light did not significantly increase collections of *An. farauti* in an earlier study by van den Hurk et al. (1997). An advantage of not using lights in mosquito traps is a reduction in nonmosquito insect by-catch. This was also observed by Carestia and Savage (1967), who found reducing by-catch simplified sorting and identification of mosquitoes. Furthermore, the light on traps may actually cause mosquito repellency, as evidenced in Vietnam, where catches of *Anopheles* and *Culex* almost doubled when light was removed from CDC light traps (Herbert et al. 1972).

The primary focus of this study was to modify current trapping systems used by the ADF to improve the quantity and survival rate of collected mosquitoes. Although the overall number of mosquitoes collected at CBTA by the FBT was not significantly different than the standard EVS and CDC light traps in this study, the CDC light trap did collect significantly more *Anopheles* than both other trap types. However, the collected mosquito survival rates were significantly higher in FBTs than the CDC light traps. Therefore, the FBT would be particularly suitable for experiments requiring living mosquitoes for studies involving dissections for estimating parity and parasite rates, and mark–release–recapture evaluations. For long-term surveillance, the FBT could be augmented with the addition of (a) larger rechargeable 12V battery (b) higher capacity CO_2_-compressed gas cylinder, and (c) a honey-soaked nucleic acid preservation cards to conduct pathogen surveillance (Hall-Mendelin et al. 2010, Van Den Hurk et al. 2012). Even without these modifications, the FBT has successfully been used for over 3 years at CBTA during both the wet and dry seasons without any issues and is currently being used by the ADF to collect mosquitoes.

## Data Availability

Data from this study are available from the Dryad Digital Repository: https://doi.org/10.5061/dryad.s1rn8pkc2 (Chow, 2023).
